# Neonatal Near Miss among Newborns of Women with Type 1 Diabetes Mellitus

**DOI:** 10.1155/2019/8594158

**Published:** 2019-07-31

**Authors:** Luiza R. Morais, Beatriz C. Patz, Felipe F. Campanharo, Patricia M. Dualib, Sue Y. Sun, Rosiane Mattar

**Affiliations:** ^1^Obstetrics Department, Universidade Federal de São Paulo (UNIFESP) Escola Paulista de Medicina, São Paulo 04021-001, Brazil; ^2^Endocrinology Department, Universidade Federal de São Paulo (UNIFESP) Escola Paulista de Medicina, São Paulo 04021-001, Brazil

## Abstract

**Objective:**

To investigate the frequency of neonatal near miss (NNM) and associate it with maternal morbidity in newborns of women with type 1 diabetes mellitus (T1DM).

**Methods:**

This was a cross-sectional retrospective study from a secondary analysis of data retrieved from medical records of pregnant women with T1DM cared at a Brazilian university hospital between 2005 and 2015. Maternal near miss (MNM) and potentially life-threatening conditions (PTLC) were classified according to the World Health Organization criteria. NNM was classified according to the Pan American Health Organization Neonatal Near Miss Working Group criteria. Association of maternal morbidity with NNM was assessed using chi-square test.

**Results:**

There were 122 newborns (NB) among 137 T1DM pregnancies. Thirty-seven NB presented NNM—incidence of 303 NNM per 1000 live births (37/122). NNM was associated with MNM (*P* < 0.001, OR (95% CI): 17.15 (1.85–159.12)). PLTC did not increase the odds of NNM (*P*=0.07; OR (95% CI): 2.1281 (0.92–4.91)). Seven newborns died, six of them from pregnancies without severe maternal morbidity. 71% of the neonatal death (5/7) occurred in malformed neonates.

**Conclusion:**

MNM was associated with NNM among women with T1DM, and PLTC, paradoxically, did not increase NNM.

## 1. Introduction

Child mortality and maternal health have been a public health concern worldwide in the last decades and were included among the eight millennium development goals (MDG) [1]. Due to the relevance of neonatal mortality worldwide, it is important to study maternal and neonatal factors that aggravate and increase it.

From 1990 to 2015, an improvement in maternal health was realized. The maternal mortality rate dropped 43.5% from 385 maternal deaths per 100,000 live births to 216 [2]. In order to improve the studies about maternal morbidity, the term maternal near miss (MNM) was defined by the WHO in 2009, denoting a woman who almost died but survived complications during pregnancy, delivery, or within the first 42 days postpartum [3]. Additionally, potentially life-threatening conditions (PLTC) were defined as an extensive category of clinical conditions that can threaten a woman's life during the same period and lead them to MNM. In parallel, a need for well-evidenced criteria for the diagnosis of neonatal near miss (NNM) cases emerged, and a definition of NNM based on consensus was proposed [4]. Current literature defines NNM as a newborn that presented severe complications during the first 28 postnatal days but survived this period despite the severity of its condition [5]. The Pan American Health Organization Neonatal Near Miss Working Group (PAHO) proposed a set of criteria to define NNM based on similar studies [6]. It is extremely important for obstetricians and pediatricians to gain in-depth knowledge on these criteria in order to identify potential NNM cases. This way, neonatal assistance can evolve and neonatal mortality may decrease.

Type 1 diabetes mellitus (T1DM) women and their newborns are formally known in literature and clinical practice as having a high risk of severe complications during the prenatal, labor, and postpartum periods as well as increased neonatal morbidity [7–10]; hence, this population is at a potentially higher risk of developing MNM and NNM. Several studies have shown the burden of poor glycemic control on fetal health and morbidity, such as a higher mortality for those with hyperglycemia [11], risk of neonatal hypoglycemia in the short term [12, 13], or neonatal mortality [14]. In the long term, there is not only higher incidence of type 2 DM and cardiovascular disorders [15] but also poor executive function and visual motor function [16]. Therefore, healthcare costs resulting from diabetic pregnant women are unmeasurable. Due to the relevance of neonatal mortality worldwide, it is important to study maternal and neonatal factors that aggravate and increase it.

We aimed to investigate the frequency of NNM in newborns of pregnant women with T1DM, verify its association with MNM and PTLC, and analyze what determines higher morbidity or mortality in this neonatal population.

## 2. Materials and Methods

### 2.1. Design and Data Collection

**Figure 1:**
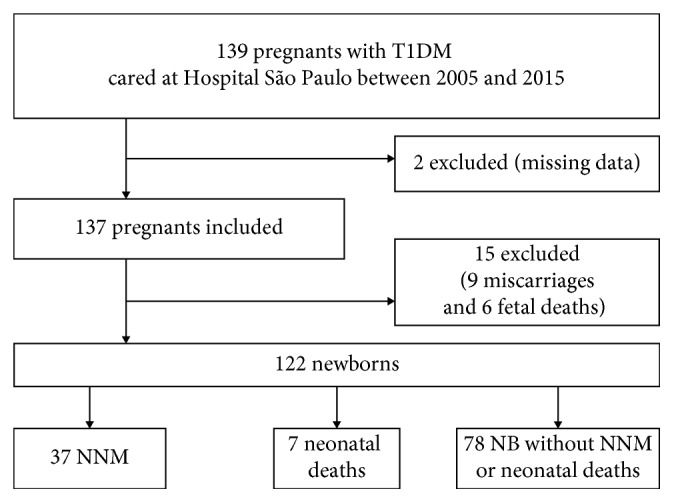
Flowchart of inclusions.

This was a cross-sectional, retrospective study conducted at São Paulo Hospital of Universidade Federal de São Paulo (UNIFESP), São Paulo, Brazil, a tertiary hospital that provides public medical care to the population through the Sistema Único de Saúde (SUS), a Brazilian unified health system. During the period of the study from January 2005 to December 2015, there were 10,070 births at this service. Eligible participants were NB of pregnant women with T1DM who delivered at Hospital São Paulo in this period whether they have received prenatal care at UNIFESP or elsewhere. Exclusion criteria referred to cases with missing data and pregnancies with fetal death or miscarriage as outcomes. The final sample included 122 newborns (Figure 1).

Data collection, held between 2016 and 2017, was conducted separately by two researchers in order to check the consistency of data extracted from medical records of pregnant women with T1DM and their newborns. Patients with missing data in clinical records were contacted by phone. We considered a ten-year study period convenient to retrieve a significant sample.

### 2.2. Definition of Variables

The variables studied for newborns were based on criteria established by PAHO [6]. Data were divided into two groups based on pragmatic and management criteria. The presence of at least one criterion classified the NB as a NNM case as established by that study.

Pragmatic criteria were as follows: birth weight <1750 g; Apgar score <7 at 5 minute, and gestational age <33 weeks. Management criteria were as follows: parenteral antibiotic therapy (up to 7 days and before 28 postnatal days), nasal continuous positive airway pressure (CPAP), intubation up to 7 days and before 28 postnatal days, phototherapy within 24 h of life, cardiopulmonary resuscitation, use of vasoactive drugs, use of anticonvulsants, use of surfactant, use of blood products, and use of steroids for the treatment of refractory hypoglycemia or surgery.

Pregnant women were classified as PLTC cases or MNM, according to the WHO criteria [3].

### 2.3. Data Analysis

Descriptive analysis of NNM and MNM criteria was conducted. Additionally, the prevalence of NNM, neonatal mortality, and early neonatal mortality were calculated. Association of MNM and PTLC with NNM was assessed using the chi-square test. A *P* value < 0.05 was considered statistically significant, and odds ratios (OR) were estimated with their 95% confidence interval. Statistical data were analyzed using Microsoft Office Excel version 2010, Microsoft, Washington, United States.

### 2.4. Ethical Aspects

The study was approved by the Institutional Review Board of UNIFESP under number 1.881.371, and the need for informed consent was waived for all included patients due to the retrospective design of the study. Also, the authors signed a document to guarantee the confidentiality and secrecy of data in order to preserve the anonymity of patients.

## 3. Results

The study included 122 newborns of pregnant women with T1DM. NNM occurred in 37 newborns, corresponding to a NNM rate of 303.7 cases per 1000 live births (37/122). There were seven neonatal deaths corresponding to a neonatal mortality rate of 57.3 deaths per 1000 live births (7/122). Six of those occurred up to 7 days, resulting in an early neonatal mortality rate of 49.1 deaths per 1000 live births (6/122).

Respiratory complications determined most cases of NNM such as using CPAP in 67.6% of cases (25/37) and intubation in 64.9% (24/37) of cases. Phototherapy was the least relevant criteria for establishing NNM followed by Apgar score <5 (Table 1).

Neonatal deaths occurred in one newborn from a pregnant woman with PTLC and six from pregnant women without any complications. Out of the 7 cases, 5 presented fetal malformation—two cases of anencephaly, one case of pulmonary hypoplasia, one case of complex congenital heart disease, and one case of genetic syndrome with multiple malformations. Two other NB died of respiratory complications.

According to the WHO criteria for maternal morbidity among 137 included pregnant women, 8 filled criteria for MNM (5.8%) and 51 for PLTC (37.2%) and 78 (56.93%) women did not present any morbidity.

Among 8 cases of MNM, there were 2 fetal deaths and 6 births. Out of the 6 NB, 5 were cases of NNM and 1 newborn had no complications. NB of pregnant women with MNM presented a significantly higher rate of NNM compared to NB of pregnancies without maternal complications (*P* < 0.001, OR (95% CI): 17.15 (1.85–159.12)) (Table 2).

PLTC did not increase the odds of NNM [*P*=0.07; OR (95% CI): 2.1281 (0.92–4.91)] (Table 3).

## 4. Discussion

In this study, NNM cases were mostly determined by respiratory interventions. Reportedly, 23% of 5.9 million deaths of children worldwide are related to neonatal hypoxia, and several conditions such as prematurity or hypoglycemia can lead to it [17]. Our findings are consistent with the fact that diabetic decompensation increases the risk of preterm delivery and fetal hypoglycemia that can lead to hypoxia [6]. Moreover, this group is at an increased risk of transient tachypnea of the NB—particularly those born by C-section [18] and persistent pulmonary hypertension [19]. In routine clinical practice, we suggest that NB of pregnant women with T1DM should be screened for respiratory complications and those who require intervention should undergo further screening for other NNM criteria.

To the best of our knowledge, the association between MNM and NNM remains unexplored. Although this study does not have the strength of a prospective follow-up study with a larger sample, an association between these two conditions could be demonstrated. OR indicated that MNM increases the odds of NNM. However, due to a wide CI, further prospective study, including a larger sample of patients, is recommended. If a pregnant woman is exposed to several systemic complications, the fetus may be inferred to be at a higher risk of hypoglycemia, prematurity, or C-section, and, consequently, at a higher risk of NNM. However, PTLC was not associated with higher rates of NNM. In this study, maternal hospitalization over 7 days determined the larger share of PLTC, most of the cases for glycemic control. In Brazil, the average annual cost per capita for a patient with T1DM is 1319.15 US dollars [20]. Our population is mostly low income, and even though the government provides free insulin supplies, not only the amount needed is usually not enough or material not available, but also patients use their supplies inappropriately. Therefore, a significant number of patients are unable to achieve proper glycemic control at home and are hospitalized for it. This group of patients with PLTC achieved proper glycemic control while hospitalized—either during pregnancy or delivery—having better neonatal outcomes and avoiding NNM. Further studies with a larger sample would also help clarify this association.

Regarding neonatal mortality, the WHO report on child mortality released in 2017 [21] estimated a global rate of newborn mortality of 19 deaths per 1000 live births in 2015. In our study, the neonatal mortality rate was 57.3 per 1000 live births. When these data are compared, it becomes clear that NB of pregnant women with T1DM are more susceptible to neonatal death.

It is a paradox that no cases of neonatal deaths were related to MNM. 71.4% of neonatal deaths in this study were related to malformation. It is known that the risk for congenital anomalies in diabetic mothers is near 7%, regardless of the type and duration of diabetes, or four times greater than in the general population [22]. A European cohort concluded that the highest rates of early neonatal mortality in newborns with malformations occurred in central nervous system anomalies or respiratory system anomalies [23] and 60% (3/5) of the cases of malformations in our study were of this kind. In our sample, we consider that MNM was not related to neonatal mortality because malformation is an independent risk factor for neonatal death, in a way that T1DM is a risk factor for malformation but does not aggravate neonatal outcomes in newborns with those malformations.

Neonatal deaths that did not occur in newborns with malformations were due to respiratory complications (2/7). Most of the patients who died required intubation or use of vasoactive drugs, and these interventions during the first week of life have been related to high mortality rates [24]. Among the 7 newborns who died, most had a birth weight <1750 g, gestational age <33 weeks, and met pragmatic criteria for NNM. A recent study conducted in Brazil with a larger sample in the general population obtained similar results, with the main cause of neonatal deaths due to respiratory disorder, low birth weight, and premature birth [25]. T1DM might have anticipated resolution of pregnancies in a way that newborns died as a complication of prematurity. However, these pregnant women probably did not present any kind of complication because the pregnancy was interrupted before further maternal complications.

This retrospective study has some limitations. The data were abstracted from existing medical records, and some of the information was missing. Another limitation is the nature of nonprobabilistic sampling, which can compromise comparison of our results with future similar studies. During our study design, we selected the 10-year period for the study since we anticipated it was going to provide us with an adequate number of patients. In the future, collecting the data prospectively and calculating the required sample size prior to the initiation of the study may allow for more better across-study results comparison.

## 5. Conclusion

The present results showed that T1DM is an important cause of NNM. It is important to offer proper neonatal care for newborns of mothers with MNM because the probability of NNM in these NB is significantly higher. NB of pregnant women with T1DM, even in a tertiary healthcare center, have very high mortality rates. However, to avoid neonatal deaths, it is advisable to offer not only prenatal care but also preconception counseling with proper glycemic control in order to diminish rates of malformation. In clinical practice, it is important to pay close attention to NB with low birth weight, low gestational age, or those requiring respiratory interventions because these factors were associated with higher neonatal mortality and NNM.

## Figures and Tables

**Table 1:** Percentage of NNM per 1000 live births among newborns of pregnant women with T1DM that met at least one criterion for NNM.

NNM criteria	*n*	% among NNM	Occurrence per 1000 live births
Surgery	8	21.6	66
Use of steroids for the treatment of refractory hypoglycemia	4	10.8	33
Parenteral antibiotic therapy	10	27.0	82
Use of vasoactive drugs	10	27.0	82
Use of surfactant	8	21.6	66
Use of anticonvulsants	7	18.9	57
Use of blood products	10	27.0	82
Cardiopulmonary resuscitation	4	10.8	33
Phototherapy within 24 h of life	1	2.70	08
Any intubation up to 7 days and before 28 days of life	24	64.9	196
Nasal CPAP	25	67.6	205
Gestational age <33 weeks	12	32.4	82
Apgar score <7 at 5 min	3	8.1	24
Birth weight <1750 g	14	37.8	115
Any (at least one of the above)	37	100.0	303
Total newborns of pregnant women with T1DM	122	—	—

NNM, neonatal near miss; CPAP, continuous positive airway pressure; T1DM, type 1 diabetes mellitus.

**Table 2:** Percentage of NNM in newborns of pregnant women with T1DM with or without MNM.

NNM	MNM (%)	Patients without any complications (%)	Risk difference (95% CI)
Yes	62.50 (*n* = 5)	17.94 (*n* = 14)	*P* < 0.001; OR: 17.15 (1.85–159.12)
No	12.50 (*n* = 1)	61.54 (*n* = 48)	

NNM, neonatal near miss; MNM, maternal near miss; CI, confidence interval; OR, odds ratio.

**Table 3:** Percentage of NNM in newborns of pregnant women with or without PLTC.

NNM	PLTC (%)	Patients without severe complications (%)	Risk difference (95% CI)
Yes	35.29 (*n* = 18)	17.94(*n* = 14)	*P*=0.0769; OR: 2.1281 (0.92–4.91)
No	56.86 (*n* = 29)	61.54 (*n* = 48)	

NNM, neonatal near miss; MNM, maternal near miss; CI, confidence interval; OR, odds ratio; PLTCs, potentially life-threatening conditions.

## Data Availability

Excel data used to support the findings of this study are available from the corresponding author upon request.

## References

[1] World Health Organization (2015). *The Millennium Development Goals Report*.

[2] World Health Organization (2019). *Trends in Maternal Mortality: 1990 to 2015*.

[3] World Health Organization (2009). *Report on the World Health Organization Working Group on the Classification of Maternal Deaths and Severe Maternal Morbidities*.

[4] Avenant T. (2009). Neonatal near miss: a measure of the quality of obstetric care. *Best Practice & Research Clinical Obstetrics & Gynaecology*.

[5] Pileggi C., Souza J. P., Cecatti J. G., Faundes A. (2010). Neonatal near miss approach in the 2005 WHO global survey Brazil. *Jornal de Pediatria*.

[6] Santos J. P., Cecatti J. G., Serruya S. J. (2015). Neonatal near miss: the need for a standard definition and appropriate criteria and the rationale for a prospective surveillance system. *Clinics*.

[7] Persson M., Norman M., Hanson U. (2009). Obstetric and perinatal outcomes in type 1 diabetic pregnancies: a large, population-based study. *Diabetes Care*.

[8] Leionen P. J., Hilesmaa V. K., Kaaja R. J., Teramo K. A. (2001). Maternal mortality in type 1 diabetes. *Diabetes Care*.

[9] Yang J., Cummings E. A., OʼConnell C., Jangaard K. (2006). Fetal and neonatal outcomes of diabetic pregnancies. *Obstetrics & Gynecology*.

[10] Ringholm L., Mathiesen E. R., Kelstrup L., Damm P. (2012). Managing type 1 diabetes mellitus in pregnancy–from planning to breastfeeding. *Nature Reviews Endocrinology*.

[11] van der Lugt N. M., Smits-Wintjens V. E. H. J., van Zwieten P. H. T., Walther F. J. (2010). Short and long term outcome of neonatal hyperglycemia in very preterm infants: a retrospective follow-up study. *BMC Pediatrics*.

[12] Metzger B. E., Lowe L. P., Dyer A. R. (2008). Hyperglycemia and adverse pregnancy outcomes. *New England Journal of Medicine*.

[13] Alemu B. T., Olayinka O., Baydoun H. A., Hoch M., Elci M. A. (2017). Neonatal hypoglycemia in diabetic mothers: a systematic review. *Current Pediatric Research*.

[14] Bental Y., Reichman B., Shiff Y. (2011). Impact of maternal diabetes mellitus on mortality and morbidity of preterm infants (24–33 weeks’ gestation). *Pediatrics*.

[15] Simeoni U., Barker D. J. (2009). Offspring of diabetic pregnancy: long-term outcomes. *Seminars in Fetal and Neonatal Medicine*.

[16] McKinlay C. J. D., Alsweiler J. M., Anstice N. S. (2017). Association of neonatal glycemia with neurodevelopmental outcomes at 4.5 years. *JAMA Pediatrics*.

[17] Teramo K. A. (2010). Obstetric problems in diabetic pregnancy—the role of fetal hypoxia. *Best Practice & Research Clinical Endocrinology & Metabolism*.

[18] Al-Agha R., Kinsley B. T., Finucane F. M. (2010). Caesarean section and macrosomia increase transient tachypnoea of the newborn in type 1 diabetes pregnancies. *Diabetes Research and Clinical Practice*.

[19] Hernandez-Diaz S., Van Marter L. J., Werler M. M., Louik C., Mitchell A. A. (2007). Risk factors for persistent pulmonary hypertension of the newborn. *Pediatrics*.

[20] Cobas R. A., Ferraz M. B., Matheus A. S. D. M. (2013). The cost of type 1 diabetes: a nationwide multicentre study in Brazil. *Bulletin of the World Health Organization*.

[21] World Health Organization (2017). *Levels and Trends in CHILD MORTALITY–Report 2017*.

[22] Bell R., Glinianaia S. V., Tennant P. W. G., Bilous R. W., Rankin J. (2012). Peri-conception hyperglycaemia and nephropathy are associated with risk of congenital anomaly in women with pre-existing diabetes: a population-based cohort study. *Diabetologia*.

[23] Groen H., Bouman K., Pierini A. (2017). Stillbirth and neonatal mortality in pregnancies complicated by major congenital anomalies: findings from a large European cohort. *Prenatal Diagnosis*.

[24] Pileggi-Castro C., Camelo J. S., Perdoná G. C. (2014). Development of criteria for identifying neonatal near-miss cases: analysis of two WHO multicountry cross-sectional studies. *BJOG: An International Journal of Obstetrics & Gynaecology*.

[25] Kale P. L., Jorge M. H. P. D. M., Fonseca S. C. (2018). Deaths of women hospitalized for childbirth and abortion, and of their concept, in maternity wards of Brazilian public hospitals. *Ciência & Saúde Coletiva*.

